# Metabolomic profiles of the liquid state fermentation in co-culture of *Eurotium amstelodami* and *Bacillus licheniformis*

**DOI:** 10.3389/fmicb.2023.1080743

**Published:** 2023-01-26

**Authors:** Yunsheng Wang, Yincui Chen, Jiankang Xin, Xianjing Chen, Tingyan Xu, Jiefang He, Zhangxu Pan, Chuanbo Zhang

**Affiliations:** Laboratory of Microbial Resources and Industrial Application, College of Life Sciences, Guizhou Normal University, Guiyang, China

**Keywords:** *Eurotium amstelodami*, *Bacillus licheniformis*, co-culture, metabolomic profiles, secondary metabolism

## Abstract

As an important source of new drug molecules, secondary metabolites (SMs) produced by microorganisms possess important biological activities, such as antibacterial, anti-inflammatory, and hypoglycemic effects. However, the true potential of microbial synthesis of SMs has not been fully elucidated as the SM gene clusters remain silent under laboratory culture conditions. Herein, we evaluated the inhibitory effect of *Staphylococcus aureus* by co-culture of *Eurotium amstelodami* and three *Bacillus* species, including *Bacillus licheniformis*, *Bacillus subtilis*, and *Bacillus amyloliquefaciens*. In addition, a non-target approach based on ultra-performance liquid chromatography time-of-flight mass spectrometry (UPLC-TOF-MS) was used to detect differences in extracellular and intracellular metabolites. Notably, the co-culture of *E. amstelodami* and *Bacillus* spices significantly improved the inhibitory effect against *S. aureus*, with the combination of *E. amstelodami* and *B. licheniformis* showing best performance. Metabolomics data further revealed that the abundant SMs, such as Nummularine B, Lucidenic acid E2, Elatoside G, Aspergillic acid, 4-Hydroxycyclohexylcarboxylic acid, Copaene, and Pipecolic acid were significantly enhanced in co-culture. Intracellularly, the differential metabolites were involved in the metabolism of amino acids, nucleic acids, and glycerophospholipid. Overall, this work demonstrates that the co-culture strategy is beneficial for inducing biosynthesis of active metabolites in *E. amstelodami* and *B. licheniformis*.

## 1. Introduction

Secondary metabolites (SMs), produced by microorganisms, are a class of low molecular weight compounds associated with a variety of biological activities. These SMs are also important sources of lead molecules in drug discovery (Chiang et al., 2009; Baral et al., 2018; Linnakoski et al., 2018; Chen et al., 2021; Wang G. et al., 2022). Since the isolation and wide use of Penicillin from *Penicillium* spp. (Fleming, 2001), researchers have isolated and identified a number of SMs with medicinal value from different microbial species. For instance, SMs isolated from *Actinomycetes* species have been important sources of antibiotics for clinical use (Shivlata and Satyanarayana, 2015). Lovastatin, a SM derived from *Aspergillus terreus* and *Monascus ruber*, is a common type of drug which is clinically prescribed to treat high cholesterol (Panda et al., 2010; Guo and Wang, 2014). In recent years, genomic sequencing and annotation data have suggested the presence of unexpectedly abundant putative SM biosynthetic gene clusters in microbial genomes (Geib and Brock, 2017; Figueiredo et al., 2021). However, previous studies have confirmed that the gene clusters for SMs are silent or exhibit very low expression levels under normal laboratory culture conditions (Chiang et al., 2011; Brakhage, 2013; Ancheeva et al., 2018), resulting in a large gap between discovered SMs and genome capability.

Microbial co-culture typically involves the cultivation of two or more strains (including fungi-fungi, fungi-bacterial, and bacterial-bacterial) in one culture vessel (Mantravadi et al., 2019), and is considered to be an excellent strategy for increasing the content and variety of SMs (Bertrand et al., 2014; Caudal et al., 2022). In the competition for living space and nutrients, microorganisms are constantly interacting with each other, which can activate the expression SM encoding gene clusters (Costa et al., 2019). Fumicyilne A was a novel SM obtained by co-culture of the soil-derived bacterium *Streptomyces rapamycinicus* and *Aspergillus fumigatus*, the production of Fumicyilne A might be attributed to the activation of the biosynthetic pathway of silent terpenoids in *A. fumigatus* (König et al., 2013). Similarly, Sung and colleagues (Sung et al., 2017) found that co-culture of Marine-Derived *Streptomyces* spp. and human pathogens resulted in enhanced production of three antibiotics, including Granaticin, Granatomycin D, and Dihydrogranaticin B. However, the use of co-culture in the activation of silent SM genes remains mostly accidental. Further, this process lacks reproducibility and predictability, which poses a significant challenge in future co-culture strategies for mining SMs.

*Eurotium amstelodami* is a filamentous fungus classified under the Phylum Ascomycota. Previous reviews and studies have shown that *E. amstelodami* can produce abundant useful SMs (Slack et al., 2009), such as Neoechinulin A, Neoechinulin B, Epiheveadride, Flavoglaucin, Auroglaucin, Isotetrahydroauroglaucin, Echinulin, Preechinulin, and Neoechinulin E. Additionally, Asperflavin, a SM isolated from marine-derived *E. amstelodami* inhibits the production of pro-inflammatory cytokines, including TNF-α, IL-1β, and IL-6 (Yang et al., 2017), which may be potentially promising lead molecules for preventing and treating inflammatory diseases. These data support the hypothesis that *E. amstelodami* is a rich resource for mining novel SMs. Similarly, *Bacillus* species are common gram-positive bacterium detected in the air (Premkrishnan et al., 2021), water (Mothe and Sultanpuram, 2016), soil (Lim et al., 2016), and fermented foods (Wu et al., 2022). Various *Bacillus* members (e.g., *Bacillus licheniformis*, *Bacillus subtilis*, and *Bacillus amyloliquefaciens*) are extensively used in the production of bioinsecticides and antibiotics due to their ability to synthesize a number of metabolites with biological activity (Xu et al., 2014; Yang et al., 2016; Shahzad et al., 2017; Mohamad et al., 2018), indicating a great potential for SM synthesis.

Many co-culture experiments involving two or more microorganisms isolated from the same habitats [e.g., soil (Wang et al., 2021), plant endophyte (Li H. T. et al., 2020), the ocean (Bao et al., 2017), and fermentation food (Liu Z. et al., 2022)] seem to show excellent effects on the induction and synthesis of SMs. In this case, microbes living and reproducing in the same ecological environment may compete for similar resources, including living space and nutrients, resulting in generating of some new compound molecules to improve viability (Knowles et al., 2022). In this work, we established a co-culture system for *E. amstelodami* and three *Bacillus* species (*B. amyloliquefaciens*, *B. licheniformis*, and *B. subtilis*) isolated from the Maotai-flavor Daqu (saccharification fermentation agent for liquor fermentation) and evaluated the inhibitory effect of different co-culture combinations on *Staphylococcus aureus*. Moreover, the metabolites (extracellular and intracellular) from the pure-culture and co-culture of *E. amstelodami* and *B. licheniformis* were analyzed and identified using a non-targeted metabolomics approach. Furthermore, we propose a potential mechanism behind the enhanced inhibition of *S. aureus* during the co-culture process.

## 2. Materials and methods

### 2.1. Strains and cultivation

*Eurotium amstelodami* GZ23, *B. licheniformis* GZ241, *B. subtilis* GZ237, and *B. amyloliquefaciens* GZ121 were isolated from Maotai-flavor Daqu produced in Renhuai City, Guizhou Province, China. According to our previous study (Wang Y. et al., 2022), *E. amstelodami* was grown on high-concentration sodium chloride MYA solid medium (malt extract 20 g, yeast extract powder 5 g, agar powder 15 g, sucrose 30 g, NaCl 170 g and water 1,000 ml) at 37°C to induce a conidia production, the appropriate concentration of conidia of *E. amstelodami* was adjusted to 1 × 10^6^ spore/mL by a blood count plate before inoculation. *Bacillus* species were inoculated in 150 ml LB liquid medium (Lysogeny Broth: 10 g/l NaCl, 5 g/l yeast extract, 10 g/l tryptone) at 37°C and 180 rpm/min for 12 h, and the cell concentration was adjusted to 1 × 10^8^ CFU/ml. To prepare the test bacteria, *S. aureus* ATCC 25923 was inoculated into 5 ml LB liquid medium and incubated overnight at 37°C while shaking at 180 rpm until a concentration of 1 × 10^6^ CFU/ml was reached.

### 2.2. Co-culture conditions

A liquid co-culture system of *E. amstelodami* and *Bacillus* spices was established. In brief, conidia of *E. amstelodami* were inoculated in 150 ml of MYA liquid medium at a volume ratio of 1:4, and cultured at 30°C on a rotary shaker at 180 rpm for 3 day. Then, the pre-cultured *Bacillus* species were added to MYA medium in the same proportion and the cultivations were continued for 7 day. For pure culture, *E. amstelodami* and *Bacillus* species were grown in 150 ml MYA liquid medium at the same inoculation ratio and incubated at 30°C for 10 day and 7 day by shaking at 180 rpm/min, respectively.

### 2.3. Antibacterial activity assay

Antibacterial activity tests were performed according to Zhang X. et al. (2022) with minor modifications. Briefly, the fermentation supernatant was collected and filtered using a 0.22 μm filter membrane to remove bacteria at the end of the cultivation. Subsequently, *S. aureus* test solution (10 μl) and 100 μl of LB liquid medium were seeded into 96-well plates, followed by addition of 80 μl of fermentation supernatant. Following incubation for 10 h at 37°C, the uninoculated sterilized MYA medium served as control. The optical density of all wells was measured using a microplate reader at 600 nm. All experiments were performed in triplicate. Antibacterial activity was calculated as a percentage as follows: Inhibition rate (%) = (*S. aureus* OD_600_ in the control – *S. aureus* OD_600_ in the test)/*S. aureus* OD_600_ in the control × 100.

### 2.4. Growth monitoring

At the end of fermentation, the pure culture broths of *E. amstelodami* or *B. licheniformis* were centrifuged at 5000 × g at 4°C for 5 min, and the supernatants were discarded. Next, cells or mycelia were harvested and washed three times with sterile water and subsequently dried at 100°C in an oven to a constant dry weight. For co-culture, the mycelia of *E. amstelodami* were separated using Whatman no.1 filter paper (Sigma-Aldrich, Darmstadt, Germany) and then washed three times in sterile distilled water. Meanwhile, due to the small size of *B. licheniformis* cells, they can easily pass through the filter paper during the filtration process and were collected after a similar centrifugation procedure to remove the supernatants. All samples were oven dried at a constant temperature before dry weight was measured. All treatments were carried out in triplicate.

### 2.5. Microscopic analysis

The ultrastructure of *E. amstelodami* and *B. licheniformis* during co-culture and pure culture was observed by scanning electron microscopy (SEM). Simply, the cultures were fixed in 5% (v/v) glutaryl glycol for 12 h at 4°C, and the excess fixative was drained off. Cells were then washed three times with phosphate buffer solution and ethanol gradient dehydration (30, 50, 70, 90 and 100% (v/v)) was performed for 10 min, followed by drying with a critical point dryer (CPD300, Leica Microsystems, Wetzlar, Germany). The samples were coated with carbon (Bal-Tec Sputter Coater SCD 005, Bal-Tec GmbH, Witten, Germany) and examined using a scanning electron microscope (UHR Nova NanoSEM 230, FEI Company, Hillsboro, OR, United States).

### 2.6. Metabolomic analysis

#### 2.6.1. Preparation of extracellular metabolome samples

The fermentation broths were centrifuged at 5,000 × g (10 min, 4°C) after cultivation and the supernatant was collected, followed by drying with LGJ-18A freeze dryer (Shanghai Hefan Instrument Co., Ltd.) to obtain a dried power, which was then stored at-80°C for subsequent analysis.

#### 2.6.2. Preparation of intracellular metabolome samples

Pure culture fermentations broths of *E. amstelodami* and *B. licheniformis* were centrifuged at 5,000 × g for 10 min at 4°C, then the supernatants were discarded, and cell pellets were washed three times with deionized water. As for the co-culture, the mycelia of *E. amstelodami* were separated by Whatman no.1 filter paper and then washed three times in sterile distilled water. Lastly, cells of *B. licheniformis* were collected by centrifugation in a frozen centrifuge. All samples were stored at-80°C for later use.

#### 2.6.3. Metabolomic detection

At the time of use, the frozen sample was ground to a fine powder in liquid nitrogen. The sample powder (50 mg) was accurately weighed into a 2 ml centrifuge tube and then thoroughly mixed with a 400 μl solution (methanol: acetonitrile = 1:1 (v:v), containing 0.02 mg/ml internal standard (L-2-chlorophenylalanine)) by using a vortex mixer. Subsequently, the mixture was fully ground using a frozen tissue lapping apparatus Wonbio-96c (containing a grinding bead with a diameter of 6 mm), followed immediately by low-temperature extraction (40 kHz, 5°C). The sample was placed at-20°C for 30 min and centrifuged for 15 min (13,000 g, 4°C). The supernatants were then transferred into the injection vials for analysis.

The extracted samples were analyzed on a UHPLC-triple TOF system from AB SCIEX coupled with a Triple TOF 5600 system. At 40°C, 10 μl of each sample were injected onto an ACQUITY UPLC HSS T3 column (2.1 × 100 mm, 1.8 μm particle size; Waters, Milford, United States) with a flow rate of 0.40 ml/min, and the total separation time for chromatographic analysis was set as 16 min. Analysis was performed following chromatographic conditions: phase A: 95% water +5% acetonitrile (0.1% formic acid in water), phase B: 47.5% acetonitrile +47.5% isopropanol +5% water (0.1% formic acid in water), *t*: 0–5 min 100% A, 0.5–2.5 min 75% A, 2.5–13 min 0% A, 13–16 min 0%A. We have previously reported the optimization of the UHPLC conditions and mass spectrometry parameters (Wang Y. et al., 2022). Further, the raw data were imported into the metabolomics software, Progenesis QI (Waters Corporation, Milford, United States), for processing and identification of characteristic peaks. The MS and MS/MS mass spectrometry information were matched to the metabolic database with the MS mass error set to less than 10 ppm, and the metabolites were identified according to the matching of secondary mass spectrometry. The main databases used were http://www.hmdb.ca, http://metlin.scripps.edu, as well as other public databases.

### 2.7. MS/MS molecular networking

The Global Social Molecular Network for Natural Products (GNPS^1^), a web-based mass spectrometry ecosystem for storing, analyzing, and sharing MS/MS spectral data, allows for visualization of datasets from different users and comparisons to all publicly available reference spectra, thereby enabling annotation of known molecules and discovery of putative analogs (Wang et al., 2016). In this study, the original disembarking data of metabolic profiling were processed in MS convert (version 3.0.10051, Vanderbilt University, Nashville, TN, USA) and uploaded separately to the GNPS platform using WinSCP software (version 5.17.3) to create a molecular network of extracellular metabolites. The main parameters for creating a classical molecular network were: Precursor ion mass tolerance of 2.0 Da and fragment ion tolerance of 0.5 Da. Edges were filtered by setting the default GNPS minimum cosine score above 0.7 and more than 6 matched peaks. Network topK of 10, minimum matched fragment ions of 2, and more than 6 matched peaks. To enhance the chemical structural information in the molecular network, the molecular networks were then performed with the MolNetEnhancer workflow, and Cytoscape software (v3.8.2) was used to visualize the interaction network.

The molecular network job (in the positive ion mode) is available at https://gnps.ucsd.edu/ProteoSAFe/status.jsp?task=cae28240c12549fcb7d50715dcc31eed.

The molecular network job (in the negative ion mode) is available at https://gnps.ucsd.edu/ProteoSAFe/status.jsp?task=81b0246e0f404468996ba0e9a58eba4e.

MolNetEnhancer network job (in the positive ion mode) is available at https://gnps.ucsd.edu/ProteoSAFe/status.jsp?task=5d47c651ac394eb6b90f6b1ac4e3af9c.

MolNetEnhancer network job (in the negative ion mode) is available at https://gnps.ucsd.edu/ProteoSAFe/status.jsp?task=32c528365e0145a5a7b6c6b2ae41f36b.

### 2.8. Statistical analysis

All experiments were performed in triplicate, and the obtained data were presented as the mean ± standard deviation. Statistical computation and plotting were conducted using Microsoft Excel 2022 and GraphPad Prism 8.0.2, respectively. The significant differences in antibacterial effects between different treatments were analyzed by one-way analysis of variance with Duncan’s test (*p* < 0.05). For UPLC-TOF-MS analysis, principal component analysis (PCA) and partial least squares discriminant analysis (PLS-DA) were performed by using ropes (R packages) version 1.6.2. Student’s t-test was used to analyze significant differences between groups. The differential metabolites between groups were identified according to the value (value >1) of variable importance in the projection (VIP), *t*-test analysis (*p* < 0.05) for the fold change (FC) analysis (FC > =1.5 or < = 0.67). Pathway enrichment analysis of the differential metabolites was performed based on the Kyoto Encyclopedia of Genes and Genomes (KEGG) database.

## 3. Results

### 3.1. Co-culture resulted in an increased antibacterial activity

This work evaluated the effect of liquid co-culture of *E. amstelodami* and three *Bacillus* species on antibacterial activity against *S. aureus*. As shown in Table 1, we observed that the cell free culture supernatant of all strains [*E. amstelodami* (20.11%), *B. licheniformis* (3.18%), *B. subtilis* (53.83%), and *B. amyloliquefaciens* (85.89%)] exhibited inhibition on *S. aureus* in pure culture. Interestingly, co-culture of *E. amstelodami* and *Bacillus* species improved the antibacterial activity against *S. aureus*, compared to pure coculture. Among them, the co-culture combination of *E. amstelodami* with *B. licheniformis* (76.71%) showed the most significant improvement in antibacterial activity, which was 3.81-fold and 24.12-fold higher than *E. amstelodami* and *B. licheniformis* pure culture, respectively. These data suggest that the co-culture strategy significantly changes the components in the liquid medium, including the degradation and transformation of some substances. As the co-culture combination of *E. amstelodami* and *B. licheniformis* showed excellent effect, we chose the combination of these two microorganisms for further analysis.

**Table 1:** Co-culture and pure culture of *E. amstelodami* and *Bacillus* species showed different inhibition effects on *S. aureus*.

Treatment	Inhibition rate (%)^a^
*E. amstelodami* pure culture	20.11 ± 0.04d
*B. licheniformis* pure culture	3.18 ± 0.01e
*B. subtilis* pure culture	53.83 ± 0.04c
*B. amyloliquefaciens* pure culture	85.89 ± 0.03a
Co-culture of *E. amstelodami* and *B. licheniformis*	76.71 ± 0.04b
Co-culture of *E. amstelodami* and *B. subtilis*	82.26 ± 0.01a
Co-culture of *E. amstelodami* and *B. amyloliquefaciens*	90.21 ± 0.01a

Data were reported as the mean value ± standard. ^a^Different letters in the same column indicated significant differences between treatments, *p* < 0.05, *n* = 3.

### 3.2. The interaction between *Eurotium amstelodami* and *Bacillus licheniformis*

**Figure 1:**
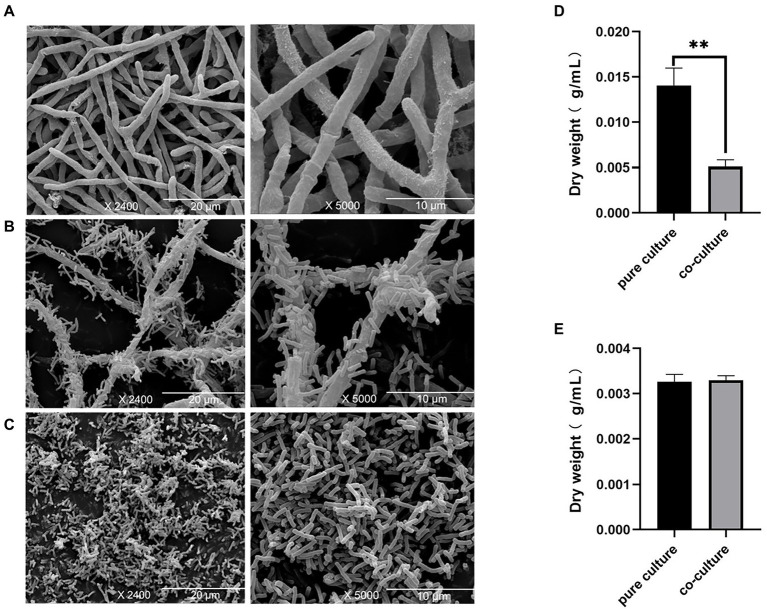
Scanning electron microscopy of *Eurotium amstelodami* mycelia in pure culture **(A)**, co-cultures **(B)** and *Bacillus licheniformis* pure culture **(C)**. Dried mycelial and cells weight of *E. amstelodami* **(D)** and *B. licheniformis* **(E)**. Data are reported as the mean value ± standard deviation of three replicates, Statistical significance (Student’s t-test) is indicated as follows: *p < 0.05.

Here, SEM was used to study the morphological characteristics of *E. amstelodami* and *B. licheniformis* during co-culture. As shown in Figures 1A,B, no significant differences in the cell morphology of *E. amstelodami* were observed between co-culture and pure culture, and similar results were obtained in *B. licheniformis* (Figure 1C). Interestingly, we found that *E. amstelodami* and *B. licheniformis* were tightly entwined together during co-culture, and few cells of individual *B. licheniformis* were present in the fermentation broth. In addition, the dry weight of *E. amstelodami* was significantly decreased by 2.75 times during co-culture compared to pure culture (Figure 1D). Conversely, *B. licheniformis* appeared to be unaffected (Figure 1E), indicating that *B. licheniformis* inhibited the growth of *E. amstelodami* during the co-culture process.

### 3.3. Extracellular metabolomic analysis of co-cultures

**Figure 2:**
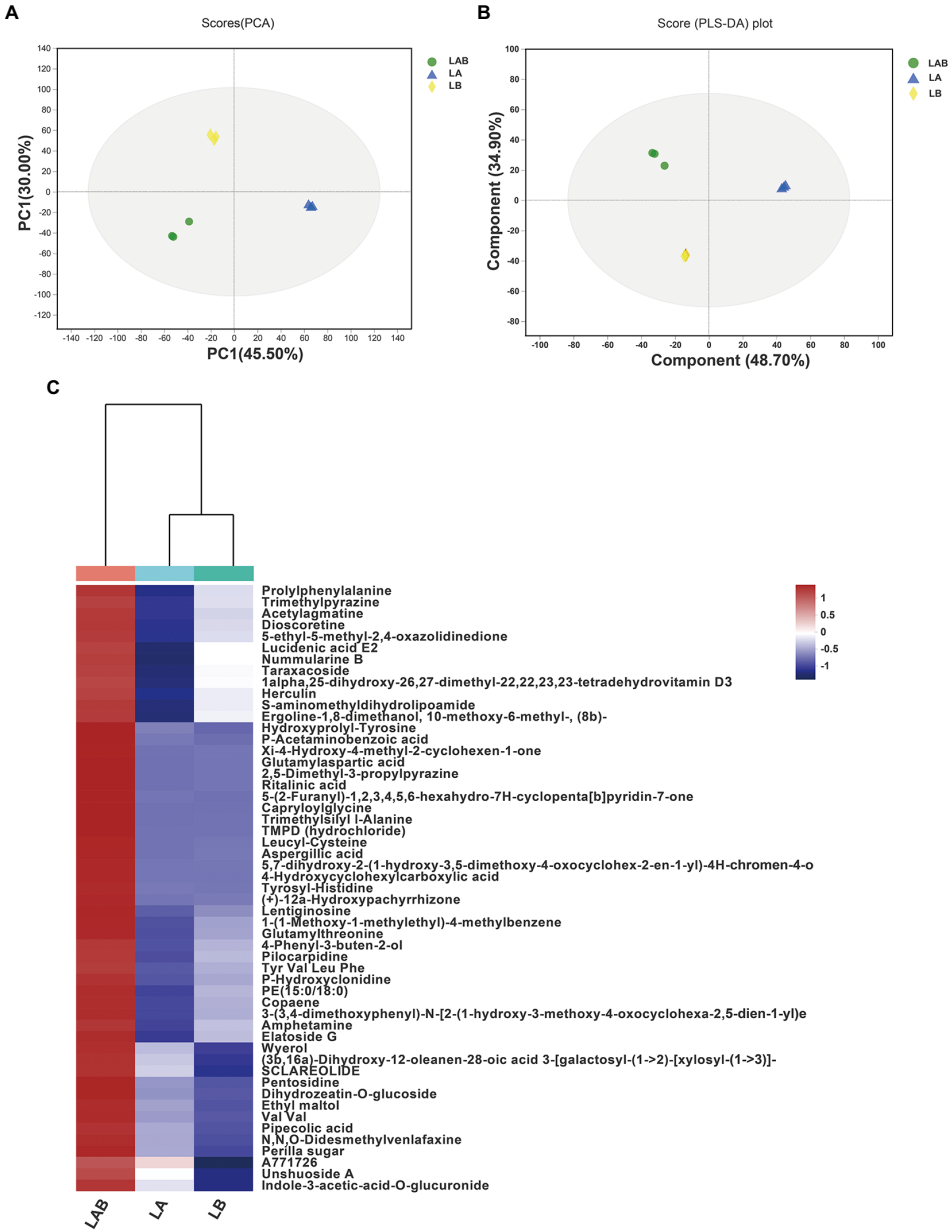
Principal components analysis (PCA) score plots of extracellular metabolites **(A)**. Partial least squares discriminant analysis (PLS-DA) score plots of extracellular metabolites **(B)**. Hierarchical clustering analysis (HCA) of the 59 extracellular metabolites only overexpressed in co-culture group and represented on a heatmap **(C)**. (LA, extracellular metabolites of *E. amstelodami* pure culture; LB, extracellular metabolites of *B. licheniformis* pure culture; LAB, extracellular metabolites of co-culture).

To decipher the mechanism behind the improvement of antibacterial activity against *S. aureus* due to co-culture, we performed metabolomic analysis for detection the extracellular metabolic differences of *E. amstelodami* and *B. licheniformis* between co-culture and pure culture. PCA analysis is an unsupervised multivariate statistical analysis method, which can reflect the overall differences and the degree of variation between the samples of each group. As shown in Figure 2A, the PCA score plots exhibited a great separation trend in extracellular metabolomic profiles among *E. amstelodami* pure culture (LA), *B. licheniformis* pure culture (LB), and co-culture (LAB), and the first and second principal components explained 45.50 and 30.00% of the total variability, respectively. To achieve and maximize distinction from different groups, the samples were grouped using a supervised partial least square discriminant analysis (PLS-DA). As shown in Figures 2B, a total of 9 samples from the 3 groups were within 95% confidence intervals. LA, LB, and LAB groups were greatly separated, and the reproducibility of samples between groups was excellent, indicating that metabolic profiles of *E. amstelodami* and *B. licheniformis* changed during co-culture. The R2X(*cum*), R2Y(*cum*), and Q2(*cum*) values obtained from PLS-DA were 0.662, 0.747, and 0.571, respectively, suggesting the model was reliable and with good prediction ability.

Based on the results of PLS-DA model, the differential extracellular metabolite features between groups were selected using a combination of VIP value >1.0, value of p (*p* < = 0.05) and fold change (FC > =1.5 or FC < =0.67). A total of 316 significantly different extracellular metabolites were found between *E. amstelodami* co-culture and pure culture (162 up-regulated and 254 down-regulated metabolites; Supplementary Table S1). At the same time, a total of 250 significantly different extracellular metabolites were obtained in *B. licheniformis* (154 up-regulated and 96 down-regulated metabolites; Supplementary Table S2). Next, we screened for the metabolites that were only overexpressed in the co-culture group. The screening criteria included (i) high contribution to sample classification in PLS-DA (VIP score > 1.5); (ii) the fold change between groups (*E. amstelodami* co-culture vs. pure culture and *B. licheniformis* co-culture vs. pure culture) was larger than 1.5; (iii) statistically significant change in the pairwise comparison between groups (*E. amstelodami* co-culture vs. pure culture and *B. licheniformis* co-culture vs. pure culture) was smaller than 0.05 (*value of p* in Student’s t-test). Finally, 53 metabolites were obtained to construct the heatmap (Figure 2C; Supplementary Table S3). We found that the contents of various amino acids, peptides, and analogs were induced to be highly expressed, including Capryloylglycine, Leucyl-Cystein, Glutamylthreonine, Prolylphenylalanine, Tyrosyl-Histidine, Pentosidine, and Hydroxyprolyl-Tyrosine. Notably, a variety of biologically active SMs were also identified, including Nummularine B, Lucidenic acid E2, Elatoside G, Aspergillic acid, 4-Hydroxycyclohexylcarboxylic acid, Copaene, and Pipecolic acid.

### 3.4. Intracellular metabolomic analysis of co-cultures

**Figure 3:**
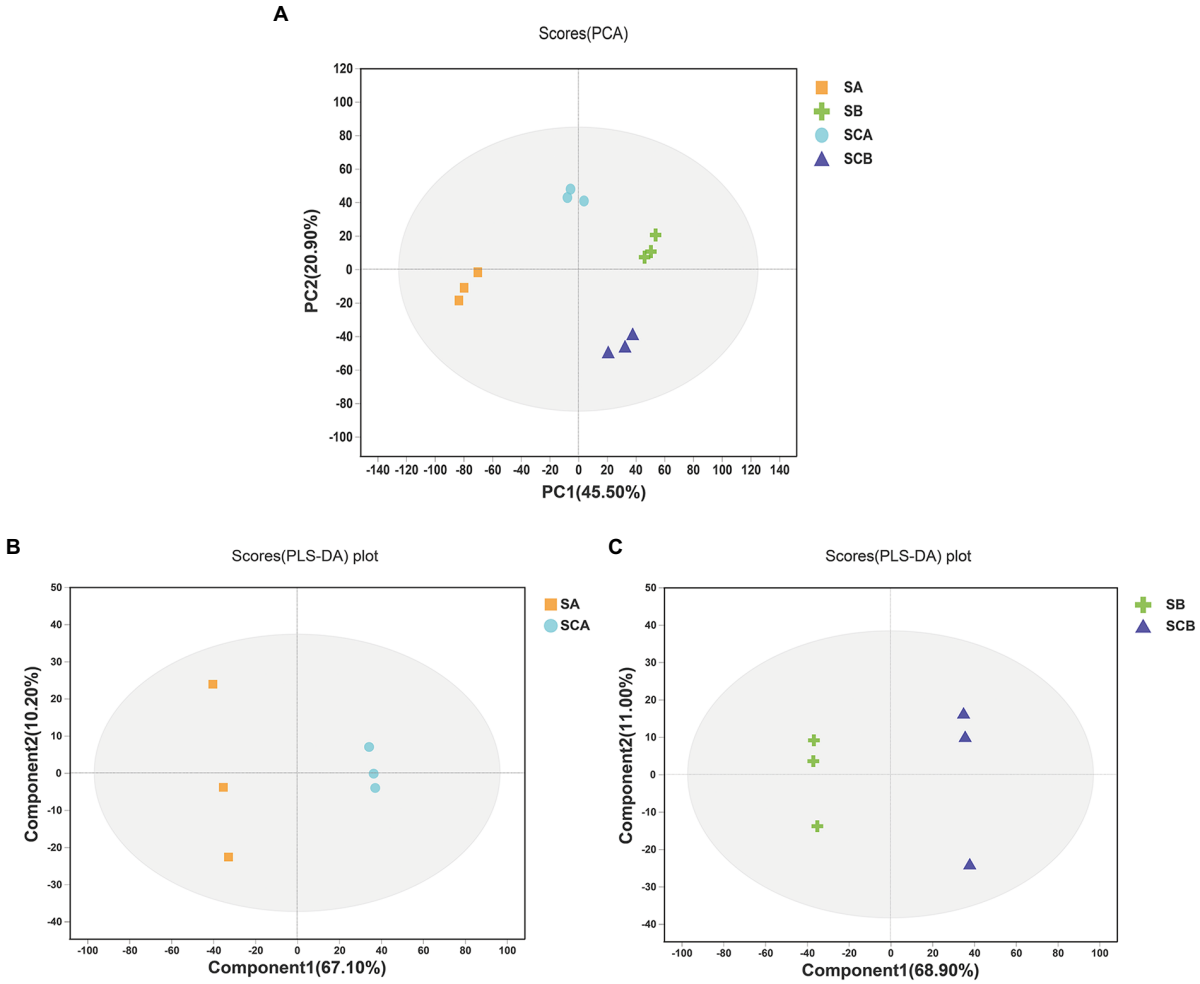
PCA score plots of intracellular metabolites **(A)**. PLS-DA score plots of intracellular metabolites between *E. amstelodami* pure culture and co-culture**(B)**. PLS-DA score plots of intracellular metabolites between *B. licheniformis* pure culture and co-culture **(C)**. (SA, intracellular metabolites of *E. amstelodami* pure culture; SCA, intracellular metabolites of *E. amstelodami* co-culture; SB, intracellular metabolites of *B. licheniformis* pure culture; SCB, intracellular metabolites of *B. licheniformis* co-culture).

The PCA score plots exhibited a good separation trend in intracellular metabolomic profiles among *E. amstelodami* pure culture (SA), *E. amstelodami* co-culture (SCA), *B. licheniformis* pure culture (SB), and *B. licheniformis* co-culture (SCB), and the first and second principal components explained 45.50 and 20.90% of the total variability (Figure 3A), respectively. As shown in Figure 3B, the intracellular metabolites of *E. amstelodami* between pure culture and co-culture were clearly distinguished in the PLS-DA model, and the reproducibility of the samples between groups was excellent. The R2X(*cum*), R2Y(*cum*), and Q2(*cum*) values obtained from PLS-DA were 0.662, 0.747, and 0.571, respectively, indicating that the model was reliable and had good prediction ability, and similar results were obtained in *B. licheniformis* (Figure 3C). These data indicated that the intracellular metabolites also dramatically changed during the co-culture of *E. amstelodami* and *B. licheniformis*.

**Figure 4:**
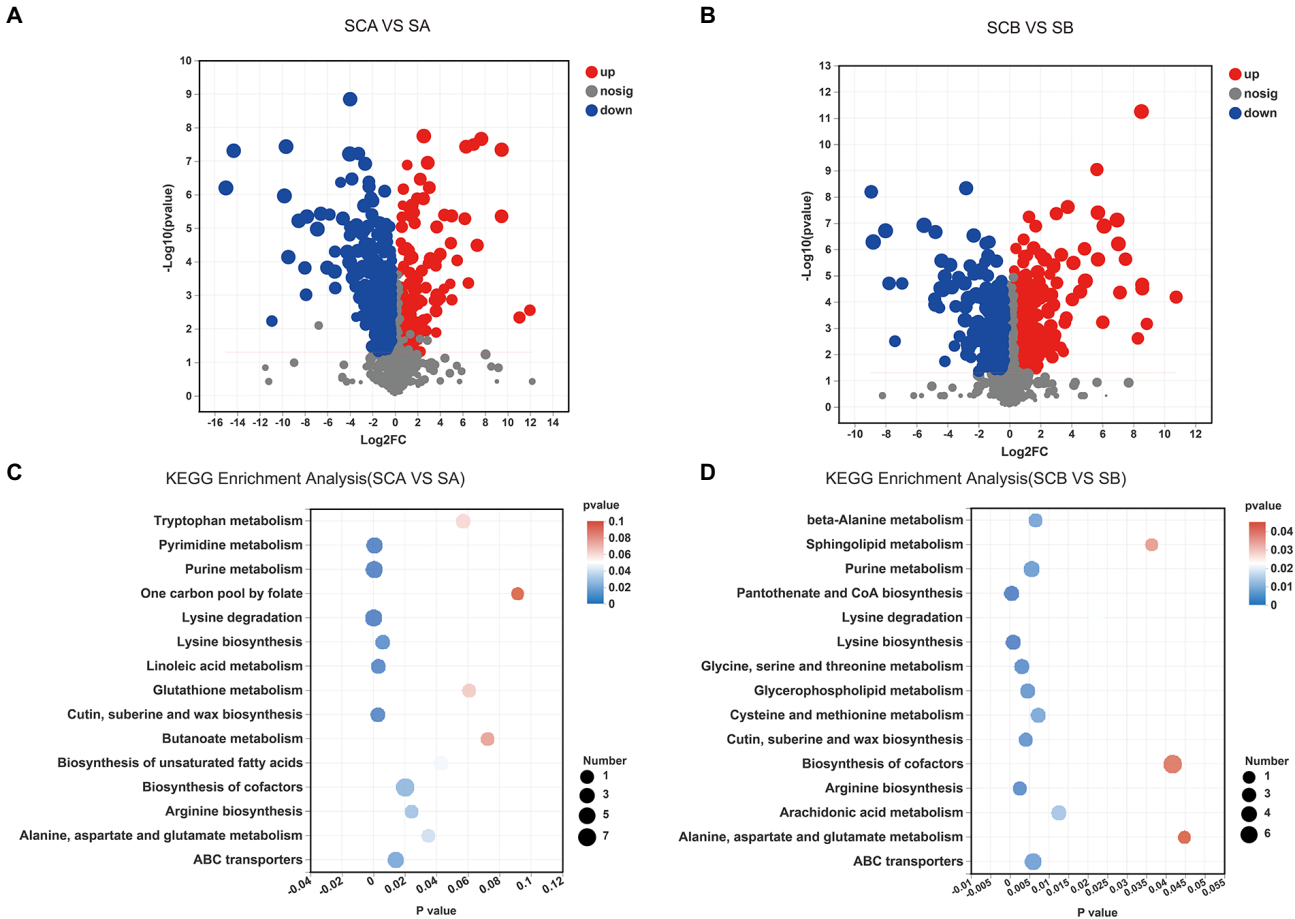
Volcano plot displaying the differences in intracellular metabolic profiles between pure culture and co-culture of *E. amstelodami* **(A)** and *B. licheniformis* **(B)**. Pathway analysis for intracellular metabolites of *E. amstelodami* **(C)**, *B. licheniformis* **(D)** pure culture and co-cultures.

A total of 397 significantly different intracellular metabolites were selected between *E. amstelodami* co-culture and pure culture (161 up-regulated and 224 down-regulated metabolites; Figure 4A; Supplementary Table S4). We found that a large number of up-regulated metabolites were annotated as amino acids, peptides, and analogs, including Capryloylglycine, Tyrosyl-Histidine, Prolylphenylalanine, Allysine, Citrulline, Glycyl-Arginine, Tyrosyl-Proline, Lysyl-Arginine, and Tyrosyl-Isoleucine. Interestingly, the contents of abundant terpenoid were enhanced during the co-culture process, such as Oleoside dimethyl ester, Cyclopassifloside II, Oleoside 11-methyl ester, (4R,6S)-p-Menth-1-ene-4,6-diol 4-glucoside, Citronellyl beta-sophoroside, 6beta-Hydroxy-3-oxo-12-oleanen-28-oic acid, 10-Hydroxymelleolide, and Ganoderic acid I. In addition, several other types of SMs (e.g., Tryptophanol, Torvoside E, Glycitin, Cholic acid glucuronide, and Norharman) were also enhanced in co-culture. Among the downregulated metabolites, various metabolites were annotated as fatty acids and conjugates, such as Palmitoleic acid, Floionolic acid, Pentadecanoic acid, 2-Isopropylmalic acid, Cyclohexaneundecanoic acid, 13-hydroxyoctadecanoic acid, and 2-Hydroxy-22-methyltetracosanoic acid. We further used the KEGG database for functional pathway enrichment analysis of differentially expressed metabolites. The results showed that the different metabolites were mainly involved in biosynthesis of cofactors (map01240), lysine degradation (map00310), purine metabolism (map00230), ABC transporters (map02010), pyrimidine metabolism (map00240), and tryptophan metabolism (map00380), which were related to the primary metabolism of microorganisms (Figure 4C).

A total of 330 significantly different intracellular metabolites were screened between *B. licheniformis* co-culture and pure culture (203 up-regulated and 127 down-regulated metabolites; Figure 4B; Supplementary Table S5). Compared to pure culture, a large number of amino acids, peptides, and analogs such as Asparaginyl-Histidine, Glycylproline, Isoleucylproline, L-Alloisoleucine, Leucyl-Cysteine, Pantetheine, Saccharopine, and Acetyl-L-tyrosine were enhanced in co-culture. Additionally, some fatty acyls and conjugates were highly expressed, including 6-(2-Hydroxyethoxy)-6-oxohexanoic acid, S-3-oxodecanoyl cysteamine, Mevalonic acid, 3,4-Methyleneadipic acid, 2-Isopropylmalic acid, and 1-Pentadecanecarboxylic acid. However, the contents of several fatty acyls and conjugates were also decreased during the co-culture process, including Floionolic acid, 2-Hydroxy-22-methyltetracosanoic acid, Physapubescin, Dodecanoylcarnitine, Sorbitan laurate, and Hexadecanedioic acid, suggesting that *B. licheniformis* required significant fatty acid metabolism in response to competition for space and nutrients in broth co-culture. The main KEGG functional pathways enriched in differential metabolites were biosynthesis of cofactors (map01240), ABC transporters (map02010), purine metabolism (map00230), glycerophospholipid metabolism (map00564), cysteine and methionine metabolism (map00270), was well as pantothenate and CoA biosynthesis (map00770; Figure 4D).

### 3.5. Molecular networking analysis

**Figure 5:**
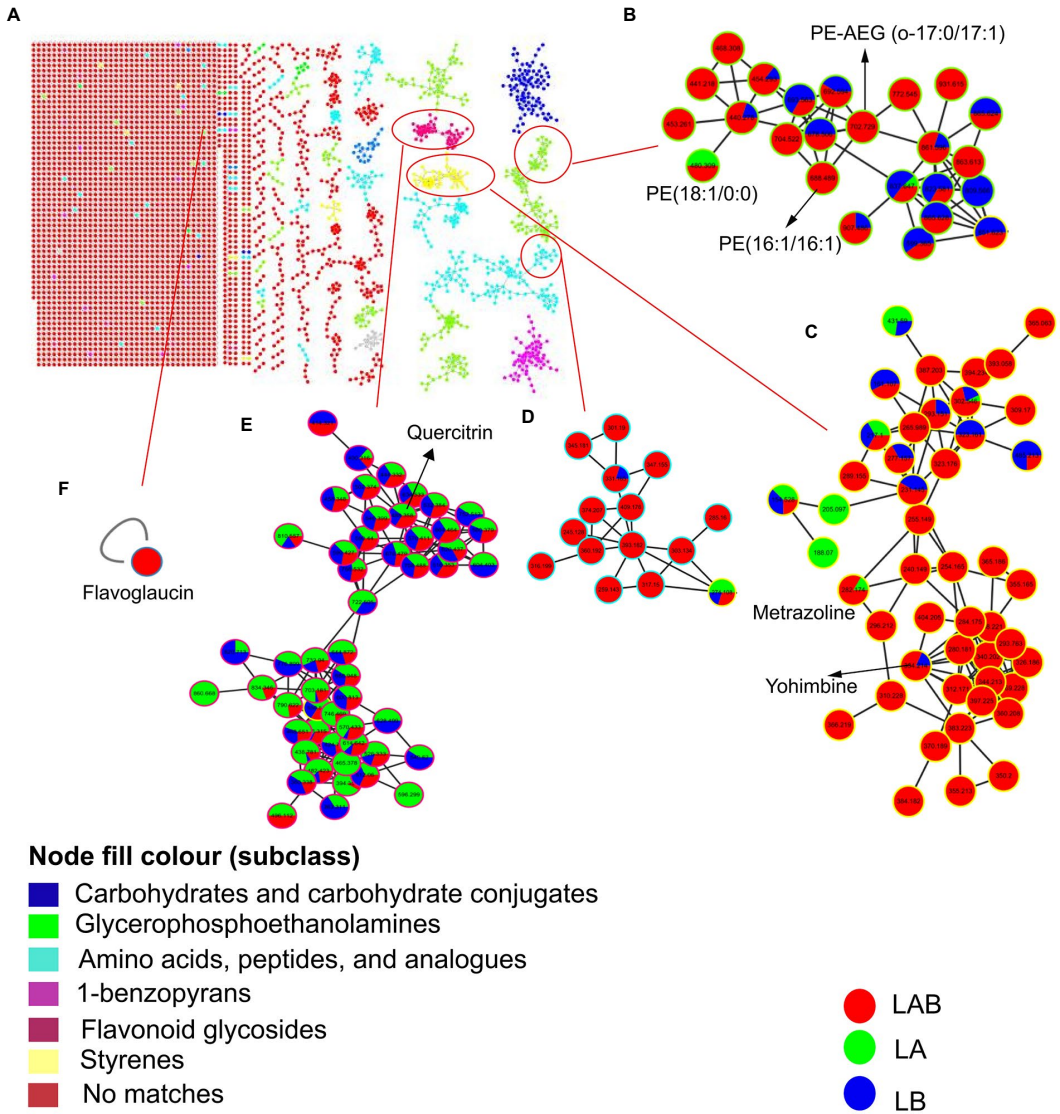
Molecular network of the MS/MS spectra for extracellular extracts of *E. amstelodami*, *B. licheniformis* pure culture and co-cultures in positive ion mode **(A)**. Chemical classification was achieved by MolNetEnhancer at the subclass level, **(B–E)** shows clusters of glycerophosphoethanolamines, styrenes, amino acids, peptides, and analogs and flavonoid glycosides, respectively. **(F)** Shows a putative flavoglaucin. Nodes represent parent ions and edge thickness corresponds to the cosine score, which represents the degree of similarity between the connected nodes. Pie ratio was determined according to scan number of spectra.

To further explore the chemical space of the extracellular metabolites produced during co-culture of *E. amstelodami* and *B. licheniformis*, we uploaded the extracellular MS–MS data to the GNPS platform for classical molecular network construction. A total of 3,787 spectra were obtained in MN, of which 457 (12.07%) consensus spectra (green node) were obtained in the *E. amstelodami* pure culture, 527 (13.92%) consensus spectra (blue nodes) were obtained for *B. licheniformis* pure culture, and 604 (15.95%) consensus spectra (red nodes) were obtained for co-culture group in the positive ionization mode. Additionally, 218 putative annotated compounds were obtained by automatic annotation of GNPS (Supplementary Table S6). Here, the MolNetEnhancer tool was used to enhance chemical structural information. As shown in Figure 5A, molecular networks of the fractions revealed the presence of clusters related to different chemical classes. The co-culture of *E. amstelodami* and *B. licheniformis* resulted in the accumulation of various metabolites in different molecular families, such as Glycerophosphoethanolamine families, including PE-AEG (o-17:0/17:1) and PE (16:1/16:1; Figure 5B); Styrenes families (a putative compound was annotated as Yohimbine; Figure 5C), and amino acids, peptides, and analogs (Figure 5D). In addition, a putative molecular family of flavonoid glycosides was annotated (Figure 5E), which exhibited no significant differences between the co-culture and pure culture group. Furthermore, Figure 5F showed a putative flavoglaucin that was detected only in the co-culture group, indicating that it was induced by co-culture.

In the negative ionization mode, A total of 4,530 spectra were obtained in MN, of which 698 (15.41%) consensus spectra were obtained in the *E. amstelodami* pure culture, 702 (15.50%) consensus spectra were obtained for *B. licheniformis* pure culture, and 741 (16.36%) consensus spectra were obtained in co-culture group. Additionally, 99 putative annotated compounds were obtained by automatic annotation of GNPS (Supplementary Table S7), less than those annotated in the positive ionization mode. As shown in Supplementary Figure S1B, a putative molecular family of benzodiazeines was annotated (Figure 5E), which exhibited no significant differences between the co-culture and pure culture group. Similarly, we observed that a putative molecular family of Glycerophosphoethanolamines (4 compounds were annotated, including PE (17:0/0:0), PE (18:2/0:0), PE (15:0/15:0) and PE (15:0/18:2)) were enhanced in the co-culture group (Supplementary Figure S1C), as well as several unannotated molecular families (Supplementary Figure S1D–E). In addition, a molecular family of Anthraquinones were identified in the *E. amstelodami* pure culture group but not in the other groups (Supplementary Figure S1F), indicating that co-culture might inhibit the production of these compounds. Interestingly, Supplementary Figure S5G showed a putative phytoceramide C2 that was detected only in the co-culture group, indicating that some new SMs were produced.

### 3.6. Co-culture affected both primary and secondary metabolism

**Figure 6:**
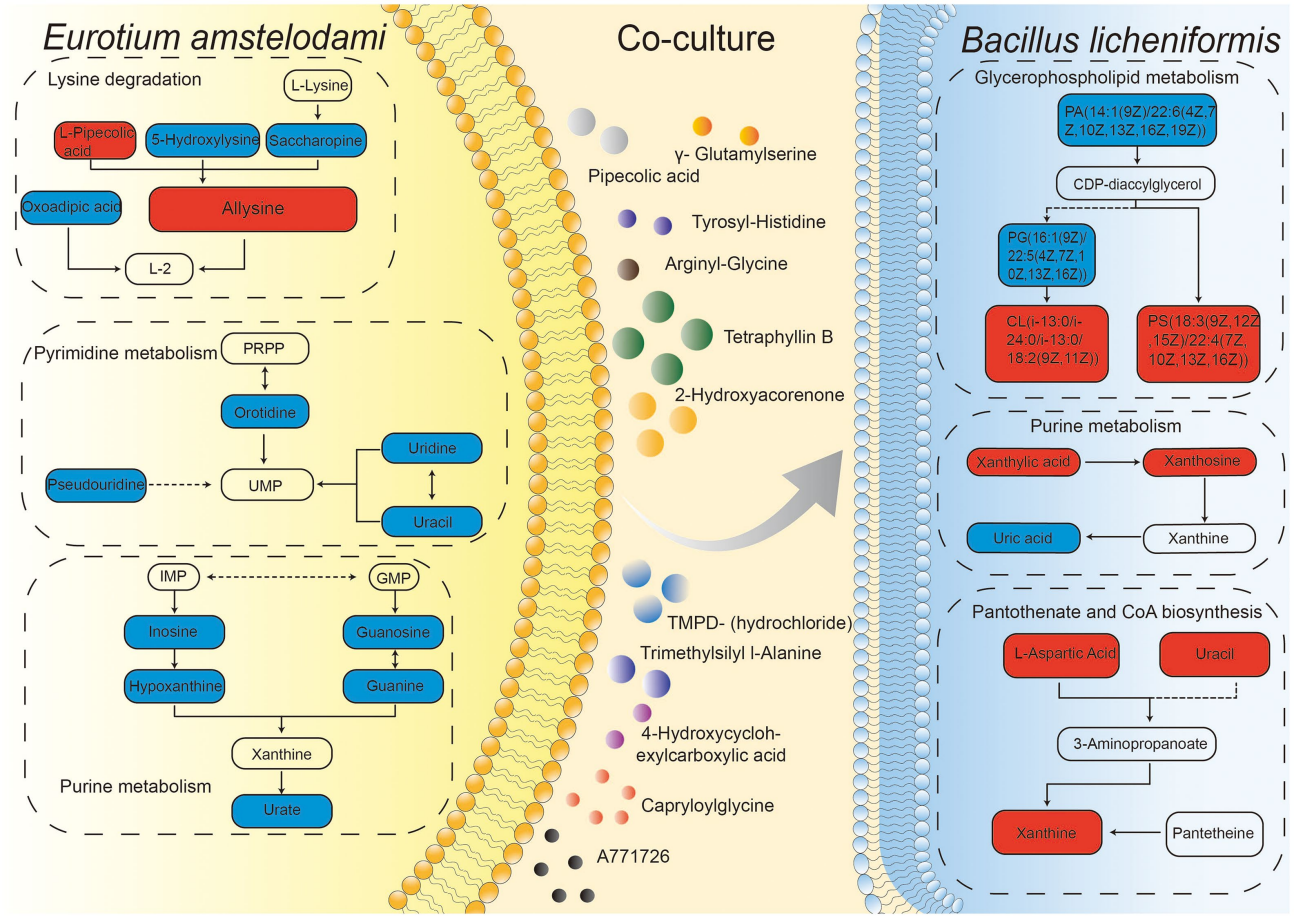
Integration map of some metabolic pathways for intracellular and extracellular metabolites of *E. amstelodami* and *B. licheniformis* co-cultures.

We summarized the changes of some intracellular and extracellular metabolites as well as metabolic pathways during co-culture of *E. amstelodami* and *B. licheniformis*. As shown in Figure 6, compared with pure culture, we found that many primary metabolites involved in amino acid metabolism and nucleic acid metabolism (pyrimidine metabolism and purine metabolism) were greatly down-regulated in *E. amstelodami*, such as oratidine, uridine, uracil, pesudouridine, inosine, guanine, guanosine, hypoxanthine, and urate, indicating that its primary metabolism was weakened. However, opposite results were obtained in *B. licheniformis*. Compared to pure culture, we observed that multiple metabolites involved in purine metabolism and pantothenate and CoA biosynthesis pathways were significantly up-regulated, including xanthylic acid, xanthosine, urcial, L-aspartic acid, and xanthine, suggesting the vigorous and active metabolism of *B. licheniformis*. Additionally, some important intermediate metabolites enriched in the glycerophospholipid metabolic pathway were differentially expressed in *B. licheniformis*. Taken together, these data suggest that co-culture conditions impact primary metabolism of these two microorganisms, resulting in large amounts of SMs being induced extracellularly.

## 4. Discussion

Our results showed that the co-culture of *E. amstelodami* and *Bacillus* species significantly improves the inhibitory effect against *S. aureus* and different co-culture combination showed different improvement effects. Many studies have demonstrated that *B. amyloliquefaciens* can produce a wide variety of SMs with desirable antibacterial activity (Shahzad et al., 2018; Mullins et al., 2020). Here, we observed that *B. amyloliquefaciens* pure cultures exhibited excellent inhibition of *S. aureus*, which was consistent with previous reports (Arguelles-Arias et al., 2009; Du et al., 2022). However, the co-culture combination of *E. amstelodami* and *B. amyloliquefaciens* achieved an impressive inhibition rate of 90.21%, it seems that the improvement was not significant compared to the pure cultures. Interestingly, the most significant improvement was observed in the co-culture of *E. amstelodami* and *B. licheniformis*, indicating that several silenced gene clusters encoding for SMs in *E. amstelodami* or *B. licheniformis* were activated, thereby enhancing antibacterial activity. The phenomenon of enhanced antibacterial activity caused by microbial co-culture with *Bacillus* species had been demonstrated in a number of studies. Li T. T. et al. (2020) found that the co-culture of *Trichoderma atroviride* and *B. subtilis* exhibited improved antifungal activity against *Fusarium graminearum*, which is the causal agent of *Fusarium* head blight in many cereal crops. Similarly, the co-culture combination of *Aspergillus sydowii* and *B. subtilis* has been shown to significantly increase inhibitory activity against *S. aureus* (Sun et al., 2022). In recent years, many research reported that *Bacillus* species was an ideal strain for establishing co-culture systems, on the one hand, *Bacillus* species, as a representative of biocontrol microorganisms, the co-culture of *Bacillus* species and other biocontrol microorganisms exhibited stronger inhibition of plant pathogenic microorganisms and promoted plant growth (Karuppiah et al., 2019; Liu H. et al., 2022), on the other hand, the co-culture of *Bacillus* species and other microorganisms could markedly increase the production of SMs contained multibiological activity (Sun et al., 2022; Zhang X. F. et al., 2022). However, due to the randomness of microbial co-culture as well as the structural complexity of SMs, many potentially promising microbial co-culture combinations remain undiscovered. Here, we propose a strategy to screen co-culture microbial combinations by targeting the improvement of antibacterial activity. Interestingly, studies have shown that changes in state of the medium (i.e., solid or liquid), composition (i.e., carbon source and nitrogen source) and culture conditions (i.e., temperature, culture length, and pH), or the addition of some trace elements might lead to improved antibacterial activity (Boruta et al., 2020; Boruta, 2021; Gao et al., 2021; Sun et al., 2022), which provided new strategies to further explore valuable SMs of the co-culture of *E. amstelodami* and *Bacillus* species.

There are three mechanisms of activating silenced gene clusters when microorganisms coexist in the same environment: (1) small-molecule secretion induced by one strain can be used as a precursor or substrate to induce the production of new SMs by another strain; (2) exogenous molecules act as a form of chemical defense to produce antibiotics or signaling molecules involved in competition between different strains; (3) direct contact activates silenced gene clusters between opposing strains resulting in the production of related SMs (Okada and Seyedsayamdost, 2017; Peng et al., 2021). In this work, the micrographs impressively demonstrated an intimate interaction between *B. licheniformis* and *E. amstelodami* in the co-culture. It is possible that this physical contact mimics the interaction pattern of the two microbes in their natural habitat. Similar results were reported previously for the co-culture of *Aspergillus oryzae* and *Zygosaccharomyces rouxii* (Liu Z. et al., 2022). Our finding was also supported by the study of Schroeckh and colleagues (Schroeckh et al., 2009) that a distinct physical contact between *Aspergillus nidulans* and actinomycetes leaded to the activation of a cryptic polyketide synthase. Interestingly, the dry weight of the mycelia significantly decreased in the co-culture of *E. amstelodami*, whereas *B. licheniformis* was unaffected. This was likely due to constant competition for nutrients and space during co-culture (Bovio et al., 2019; Liu Z. et al., 2022), and *E. amstelodami* seems to be a weak competitor. Notably, members of *Bacillus* species are well-known producers of lipopeptides that can inhibit the growth of some fungi (Hossain et al., 2016; Lax et al., 2019). We speculated that *B. licheniformis* produced lipopeptides that inhibited the growth of *E. amstelodami*, which merits further investigation.

Since microbial co-culture involves interactions between different species or the same species but different strains, as well as formed metabolites that are more complex and diverse (Adnani et al., 2015). These data underscore the importance of efficient detection methods of metabolites in co-culture. In the current study, UHPLC-QTOF-MS/MS was used to accurately detect the differences in metabolites (intracellular and extracellular) between *E. amstelodami* and *B. licheniformis* in co-and pure culture. Based on the PCA and PLS-DA results, intracellular and extracellular metabolites were clearly separated between the different groups. Similar findings were confirmed in a variety of microbial co-culture combinations (Oppong-Danquah et al., 2018; Detain et al., 2022), indicating that co-culture results in significant changes in compound diversity. This conclusion was confirmed by differential metabolite screening. The content of a large number of biologically active SMs, including Nummularine B, Lucidenic acid E2, Elatoside G, Aspergillic acid, 4-Hydroxycyclohexylcarboxylic acid, Copaene, and Pipecolic acid, were significantly increased in the co-culture group. Importantly, Nummularine B, a cyclic peptide alkaloid, exhibited effective inhibitory effects on porcine epidemic diarrhea virus (PEDV) (Kang et al., 2015). Lucidenic acid E2 is a triterpenoid, which widely exists in *Ganoderma lucidum* and shows certain anti-tumor effects (Iwatsuki et al., 2003; Che et al., 2017). Elatosides G, a saponin, was found to exhibit potent hypoglycemic activity in the oral glucose tolerance test in rats (Yoshikawa et al., 1995). Aspergillic acid, a derivative of natural pyrazines, is an antibiotic substance produced by *Aspergillus flavus* (Woodward 1947). Copaene is a tricyclic sesquiterpene, which has been shown to increase antioxidation in human lymphocyte cultures (Türkez et al., 2014). Pipecolic acid is an important precursor of a number of useful microbial SMs, and Piperinic acid-derived fractions have been proposed as an essential bioactivity of certain microbial natural products in pharmaceutical applications (He, 2006). Ulteriorly, several typical molecular clusters of SMs were identified based on a molecular network tool in GNPS, including flavonoid glycosides, anthraquinones and harmala alkaloids. Interestingly, several compounds, which were only annotated or had relatively high content in co-culture, such as PE-AEG (o-17:0/17:1), PE (16:1/16:1), yohimbine, flavoglaucin, and phytoceramide C2. Flavoglaucin is an antioxidant produced by molds used in fermented foods (Miyake et al., 2010). Notably, we found a large number of unannotated molecular families both in the co-culture and pure culture, indicating a tiny fraction of this vast chemical space had been discovered. Although current research revealed that a large number of SMs were induced during co-culture process, the specific SMs that play an inhibitory role remain unknow. In the future, the isolation of SMs may be exciting and interesting to know which compounds exert antimicrobial effects and understand their production mechanisms.

Amino acid metabolism plays an important biological function in microbial growth and in the synthesis of SMs (El Hajj Assaf et al., 2020; Subba et al., 2022). We found that the content of amino acids and derivatives were significantly increased in the fermentation broth during the process of co-culture, such as allysine, leucyl-lysine, prolylphenylalanine, trimethylsilyl L-alanine and arginyl-glutamine. According to Wu et al., co-culture of *B. amyloliquefaciens* ACCC11060 and *Trichoderma asperellum* GDFS1009 resulted in a significant enhancement of amino acids (Wu et al., 2018), including d-aspartic acid, L-allothreonine, L-glutamic acid, L-histidine, L-isoleucine, L-leucine, L-proline, and L-serine, which was quite similar to our result. Further, this provides a theoretical basis for exploring the production of large amounts of amino acids by microbial fermentation.

Nucleic acid (purine metabolism and pyrimidine metabolism) metabolism plays an important role in the synthesis of microbial genetic material, energy supply, metabolic regulation, and as messenger molecules, as well as in the formation of coenzymes (Rang et al., 2020). Here, we observed that several important intermediate metabolites were dramatically decreased in *E. amstelodami* co-cultures, indicating that the nucleotide metabolism of *E. amstelodami* was attenuated, this was consistent with our observation that the growth of *E. amstelodami* decreased.

Glycerophospholipids are important components of cell membranes (Sohlenkamp and Geiger, 2016; Qian et al., 2022). Previous studies have shown that glycerophospholipid metabolism plays an especially important role in the response to extracellular stress (Lopalco et al., 2017; Zai et al., 2017). Here, many important intermediate metabolites of glycerophospholipid metabolic were differentially expressed in the co-culture process (both *E. amstelodami* and *B. licheniformis*), suggesting that these two microbes activated the glycerophospholipid metabolic pathway in coping with co-culture stress.

In summary, the co-culture combination of *E. amstelodami* and Bacillus species could significantly improve antibacterial activity against *S. aureus*. Here, we demonstrate that the co-culture strategy successfully induced a large number of biologically active SMs. To the best of our knowledge, this is the first metabolomics-based report of metabolite profiles of *E. amstelodami*. These results provide novel insights into the selection of good co-culture combinations, as well as activation of microbial SM synthesis silencing gene clusters.

## Data availability statement

The original contributions presented in the study are included in the article/Supplementary material, further inquiries can be directed to the corresponding author.

## Author contributions

CZ: conceptualization, supervision, revision, validation, and investigation. YW: experiment, software, and writing—original draft preparation. YW, YC, XC, JH, and JX: resources. YW, YC, and TX: data curation and project administration. YW and YC: data analysis. CZ and YW: visualization. All authors have read and agreed to the published version of the manuscript.

## Funding

This study was supported by the National Natural Science Foundation of China (Nos. 31860438 and 81760688).

## Conflict of interest

The authors declare that the research was conducted in the absence of any commercial or financial relationships that could be construed as a potential conflict of interest.

## Publisher’s note

All claims expressed in this article are solely those of the authors and do not necessarily represent those of their affiliated organizations, or those of the publisher, the editors and the reviewers. Any product that may be evaluated in this article, or claim that may be made by its manufacturer, is not guaranteed or endorsed by the publisher.

## Footnotes

^1^ https://gnps.ucsd.edu/
